# Cargo binding promotes KDEL receptor clustering at the mammalian cell surface

**DOI:** 10.1038/srep28940

**Published:** 2016-06-29

**Authors:** Björn Becker, M. Reza Shaebani, Domenik Rammo, Tobias Bubel, Ludger Santen, Manfred J. Schmitt

**Affiliations:** 1 Molecular and Cell Biology, Department of Biosciences and Center of Human and Molecular Biology (ZHMB), Saarland University, D-66041 Saarbrücken, Germany; 2 Department of Theoretical Physics, Saarland University, D-66041 Saarbrücken, Germany

## Abstract

Transmembrane receptor clustering is a ubiquitous phenomenon in pro- and eukaryotic cells to physically sense receptor/ligand interactions and subsequently translate an exogenous signal into a cellular response. Despite that receptor cluster formation has been described for a wide variety of receptors, ranging from chemotactic receptors in bacteria to growth factor and neurotransmitter receptors in mammalian cells, a mechanistic understanding of the underlying molecular processes is still puzzling. In an attempt to fill this gap we followed a combined experimental and theoretical approach by dissecting and modulating cargo binding, internalization and cellular response mediated by KDEL receptors (KDELRs) at the mammalian cell surface after interaction with a model cargo/ligand. Using a fluorescent variant of ricin toxin A chain as KDELR-ligand (eGFP-RTA^H/KDEL^), we demonstrate that cargo binding induces dose-dependent receptor cluster formation at and subsequent internalization from the membrane which is associated and counteracted by anterograde and microtubule-assisted receptor transport to preferred docking sites at the plasma membrane. By means of analytical arguments and extensive numerical simulations we show that cargo-synchronized receptor transport from and to the membrane is causative for KDELR/cargo cluster formation at the mammalian cell surface.

Sensing of and responding to extracellular stimuli is an intrinsic property of eukaryotic cells to tightly regulate essential basic processes such as proliferation, migration, neurotransmission, or even immune defense^1^,^2^,^3^,^4^,^5^,^6^. In particular plasma membrane (PM) receptors, e.g. G-protein coupled receptors (GPCRs), play an important role in recognizing extracellular ligands, such as peptide hormones or drugs, and subsequently transducing the exogenous signal into a cellular response^7^. In this context, a series of cell surface receptors, including EGF and T-cell receptors as well as receptors that are parasitized by certain A/B toxins or viruses for endocytic internalization, are known to cluster in dynamic membrane nano-domains allowing cells to tune signaling efficiency and ligand sensitivity, or control protein interactions^7^,^8^,^9^,^10^,^11^,^12^. Since various human diseases are directly linked to abnormalities in membrane-receptor distribution and/or activation, it is important to understand the underlying mechanistic principles responsible for receptor clustering and dynamic reorganization to develop potential strategies for a therapeutic treatment^6^,^8^,^13^.

To address such essential biophysical aspects in receptor biology, we focused on mammalian KDEL receptors (KDELRs) at the cell surface that we and others have shown to be responsible for the sensing and binding of KDEL-cargo and KDEL-bearing A/B toxins^14^,^15^,^16^,^17^. Besides having a central function in the retrieval of luminal proteins of the endoplasmic reticulum (ER) and in KDEL-cargo uptake from the cell surface, KDELRs are also known to act as GPCRs in the regulation of gene expression. The loss of KDELR1 has been recently demonstrated to cause lymphopenia and a failure in controlling chronic viral infections^18^,^19^,^20^. Because of the biomedical importance of KDELRs at the mammalian cell surface we addressed this aspect in more detail and aimed to answer the following questions: (i) How are KDELRs distributed in the PM and how does cargo binding affect receptor dynamics and distribution at the cell surface? (ii) How do cells respond to cargo binding and what is the underlying cellular mechanism? In contrast to the majority of studies on receptor clustering that either focused on biological or on theoretical aspects, we here followed a combined experimental, computational, and theoretical approach to dissect and modulate cargo binding, internalization and cellular response mediated by KDELRs at the mammalian cell surface. We thereby demonstrate that cargo binding induces dose- and temperature-dependent receptor clustering at and internalization from the PM that is accompanied and counteracted by microtubule-assisted anterograde receptor transport to distinct docking sites at the membrane. Based on the results of extensive Monte Carlo simulations and analytical arguments we disentangle the effects of surface dynamic processes from those of cargo-synchronized anterograde KDELR transport along the microtubule network towards and from the PM, and verify that the statistical properties and temporal evolution of the receptor cluster-size distribution is mainly induced and controlled by the later process.

## Results

KDELRs represent transmembrane proteins which recognize and bind soluble residents of the ER containing a C-terminal retention motif (KDEL or KDEL-like) to prevent escape from the secretory pathway^20^,^21^. Recent studies however demonstrated that KDELRs are not restricted to ER and Golgi compartments but also localize in the PM where they bind KDEL-cargo such as mesencephalic astrocyte-derived neurotrophic factor (MANF)^17^ and internalize microbial A/B toxins such as the HDEL-bearing K28 virus toxin^14^,^15^,^16^. Until now, however, it is unknown what mechanistically happens after a potential H/KDEL-cargo has bound to the pool of PM localized KDELRs. In addition to the equilibrium between anterograde receptor delivery to and internalization from the plasma membrane, receptor clustering as well as lateral membrane diffusion in response to ligand binding could play a key role in determining the total amount of KDELRs at the cell surface, similar to how EGFR (epidermal growth factor receptor) and AChR (acetylcholine receptor) control ligand sensitivity and activate signaling pathways^8^,^9^,^22^.

### Design and biological activity of a model KDELR cargo

**Figure 1:**
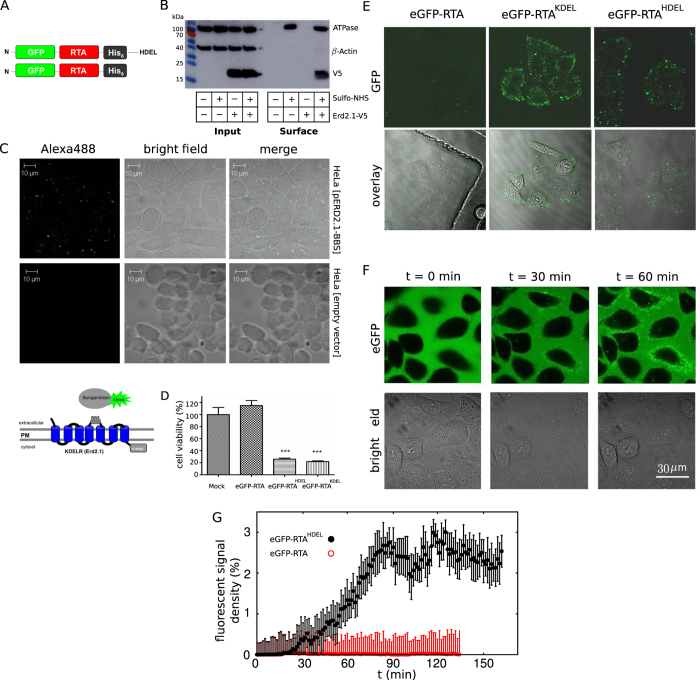
H/KDEL-cargo binding to the mammalian cell surface induces receptor cluster formation. (**A**) (top) Schematic outline of the fluorescent model cargo eGFP-RTA^HDEL^ consisting of the cytotoxic A-subunit of ricin (RTA), mammalian enhanced GFP (eGFP) and a C-terminal (His)_6_-Tag for purification. (bottom) eGFP-RTA lacking a KDELR binding motif served as negative control. (**B**) Cell surface biotinylation of mammalian KDELR1. HeLa cells were transiently transfected with KDELR1 (Erd2.1-V5 (+)) or an empty vector (−) and cultivated for 48 h. Cell surface proteins were biotinylated by treatment with (+) or without (−) Sulfo-NHS-SS-Biotin and purified with streptavidin beads. Whole cell lysates (input) served as control to determine the total amount of Erd2.1-V5 (detected with anti-V5), while *β*-actin and Na+/K+ ATPase served as cytosolic and plasma membrane marker proteins, respectively. Membrane fraction (surface) illustrates the total fraction of proteins at the cell surface. (**C**) (bottom) Schematic outline of *α*-bungarotoxin (Btx) cell surface binding. HeLa cells expressing a KDELR variant in which a Btx binding site (BBS) was inserted between positions T114 and P115 of c-myc-tagged KDELR1 (Erd2.1) were treated with Alexa488-labeled *α*-Btx. As Btx is incapable to cross the mammalian PM, any physical interaction between Btx and BBS can only occur if KDELR1 is present in the PM. (top) Confocal laser scanning microscopy of HeLa cells transfected with pERD2.1-BBS-cmyc or an empty vector control and treated with 10 *μ*g/ml Alexa488-labeled *α*-Btx. (**D**) *In vivo* toxicity of eGFP-RTA^H/KDEL^ against HeLa cells. Cell viability (N = 3, n = 5 replicates) was determined after 48 h in the presence or absence of 160 *μ*g/ml of the indicated RTA variant (Mock, PBS buffer). Mean values and standard deviations are displayed (***P < 0.001, t test). (**E**) Fluorescence microscopy of cargo binding at the cell surface. HeLa cells were treated with 160 *μ*g/ml eGFP-RTA^H/KDEL^ or eGFP-RTA for 5 min and cargo binding was analyzed after 10 washing steps. (**F**) Live cell imaging (45 frames/h) of HeLa cells treated with 160 *μ*g/ml eGFP-RTA^HDEL^. Three representative time points (0, 30, 60 min) are shown. (**G**) Temporal evolution of the density of cargo signals at the surface of HeLa cells. The accumulation of fluorescent signals is shown after treatment with 160 *μ*g/ml eGFP-RTA^HDEL^ or eGFP-RTA. The symbols represent the optimal signal-to-noise ratio in image analysis. The error bars reflect the variation range of signal intensity for different threshold values of image analysis parameters. The functional form only weakly depends on the choice of the threshold values.

KDELR cluster formation at the mammalian cell surface in response to cargo binding was analyzed and visualized on a model cargo by using a GFP-tagged variant of the cytotoxic A-subunit of ricin (RTA) extended by a C-terminal H/KDEL motif (Fig. 1A). Using cell surface biotinylation, we were able to detect KDELR1 at the cell surface of mammalian cells (Fig. 1B), in agreement with recent studies in which a pool of KDELR1 was observed at the PM of neuroblastoma cells^17^. Cell surface localization of KDELR1 was also analyzed by adapting an imaging assay originally designed to confirm PM-localization of AMPA receptors through binding of the snake venom *α*-bungarotoxin, Btx^23^,^24^. For imaging analysis at the cell surface, a Btx binding site (BBS) was inserted into an extracellular loop of mammalian Erd2.1 (KDELR1) and subsequently used to visualize physical Btx/KDELR interaction at the PM (Fig. 1C, bottom). Extracellular binding of Alexa488-labeled Btx to the modified and *in vivo* functional KDELR1 variant containing an Btx binding motif in an extracellular loop of the receptor further supported the biotinylation data and likewise demonstrated that a minor but significant number of receptors localizes in clusters at the PM of HeLa cells (Fig. 1C, top). The recombinantly expressed and purified cargo protein eGFP-RTA^H/KDEL^ remained toxic to HeLa cells (see Fig. 1D and Supplementary Fig. S1), confirming earlier observations that the addition of a C-terminal H/KDEL motif to RTA enhances its *in vivo* toxicity^25^,^26^. In contrast to the negative control of eGFP-RTA lacking a C-terminal KDELR binding site, eGFP-RTA^H/KDEL^ rapidly bound to the cell surface within seconds to minutes, indicating that a fraction of KDELRs at the PM is responsible for ligand binding (Fig. 1E and Supplementary Fig. S2). This is further supported by the similar behavior seen in cell surface cargo clustering in response to eGFP-RTA^HDEL^ addition and KDELR1 pattern formation after Btx binding (Fig. 1C). As analogous KDELR1/cargo clustering was also observed in different cell lines (such as HEK-293T and RAW-Blue), KDELR-mediated cargo binding at the PM is not restricted to just a single cell type but rather seems a general phenomenon in mammalian cells (Supplementary Fig. S3). Immunostaining of non-permeabilized HeLa cells as well as binding studies at 4 °C further demonstrated that eGFP-RTA^HDEL^ signals are indeed present at the plasma membrane and, thus, not restricted to signals of internalized KDELR/cargo complexes (Supplementary Figs S2B and S4). In addition, increased toxicity of eGFP-RTA^H/KDEL^, visible intracellular fluorescent signals, especially after longer incubation (>6 h) in live cell imaging, and the observed endocytic uptake of KDELR1 in the modified/reversed biotinylation experiment further indicate that eGFP-RTA^H/KDEL^ is indeed internalized from the mammalian cell surface (see Fig. 1D,G, Supplementary Fig. S5 and Supplementary Movies S1 and S2).

Furthermore, live cell imaging of cells loaded with eGFP-RTA^HDEL^ (see Fig. 1F) identified a strict time-dependent accumulation of fluorescent cargo signals at the PM which was absent in control cells treated with eGFP-RTA lacking a KDELR binding motif (Fig. 1H). Interestingly, the development of fluorescent signals/clusters of eGFP-RTA^HDEL^ at the cell surface occurred in distinct phases: Initially, the system remained relatively inactive for a short time (*t* < 20 min). After this transient regime, an exponential growth was observed, which eventually saturated at *t* > 80 min. The observed huge fluctuations of the accumulated receptor density at the PM is a signature of the stochasticity of the underlying nonequilibrium process, where the system ultimately reaches a balance between the loss of surface receptors due to endocytosis and gain by recycling them.

### Adaptive Monte Carlo simulations of KDELR/cargo dynamics at the cell surface

**Figure 2:**
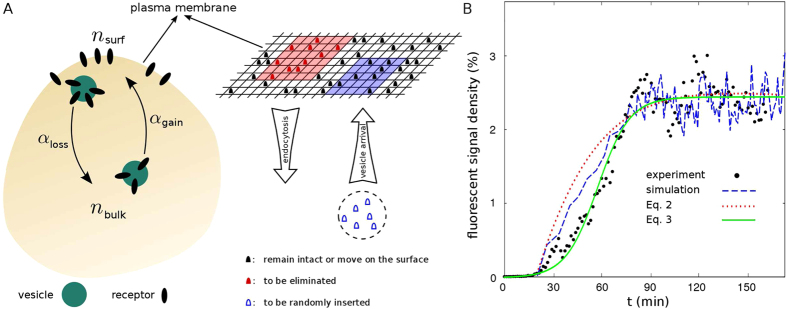
(**A**) Schematic representation of (left) the minimal model of receptor cycle between the PM and endosomes, and (right) the simulation method. An example of a randomly chosen area for endocytosis (vesicle arrival) is marked in red (blue). Possible scenarios for the evolution of the surface receptor population during the next simulation step are shown. (**B**) Time evolution of the density of accumulated cargo at the cell surface. A comparison is made between experimental data, simulation results (a single realization), and the analytical expressions Eqs 2 and 3. The dotted line indicates the analytical prediction via Eq. 2 for *α*_gain_ = *α*_loss_ = 1.3 × 10^−4^ s^−1^. The starting time of simulations and analytical expression 2 is shifted to take into account the initial inactive regime in experiments.

Aiming at better understanding the *in vivo* observed KDEL/cargo interactions at the cell surface, we performed extensive Monte Carlo simulations which shed light on the underlying mechanisms of receptor clustering at the cell membrane. We modeled the cell membrane as a lattice with a spacing of the size of receptors (~10 nm)^27^,^28^ and periodic boundary conditions (Fig. 2A). Each lattice site can be occupied by at most one receptor which is either liganded or unliganded. The membrane size (4 × 10^6^ lattice sites) is comparable to that of a typical HeLa cell. Assuming that the frequency, spatial extent, and target region of endocytosis and recycling of receptors are independent stochastic events, we introduced asymmetric rates of endocytosis and vesicle arrival, and chose a random target region on the membrane for each event. Considering the normal size of clathrin-coated vesicles to be in the range of 50 to 100 nm^29^, we allowed the extent of events to vary within 5 × 5 to 10 × 10 lattice sites. An endocytosis event leads to elimination of all receptors within the affected zone. The number of receptors carried by an incoming vesicle was chosen randomly from 0 to the maximum capacity of that vesicle and distributed randomly within the targeted zone on the PM upon availability of empty sites. One may also switch on the receptor surface dynamics, including lateral membrane diffusion and receptor-receptor interactions. Starting with an initial random configuration of receptors on the lattice, the surface density evolves and finally reaches a nonequilibrium steady state by balancing the receptor gain and loss. There are density fluctuations in the steady state due to the stochastic origin of endocytosis and vesicle arrival events. As shown in Fig. 2B, the experimental data could be qualitatively reproduced in simulations by tunning the initial density at the PM and the gain and loss rates. Notably, the amplitude of steady-state oscillations in simulations obtained for a single realization is comparable to the experimental data.

### Models for receptor cycle

We first developed a minimal theoretical model for loss and gain of receptors during endocytosis and recycling back to the surface. Assuming that the total number of receptors in the cell is conserved within our experimental time window, the fractions of receptors at the cell surface *n*_surf_ and inside the cell *n*_bulk_ are related as *n*_surf_ + *n*_bulk_ = 1. Denoting the endocytosis and vesicle arrival rates, respectively, with *α*_loss_ and *α*_gain_, the evolution of the average fraction of receptors at the plasma membrane *n*_surf_(*t*) in a simple form can be described as


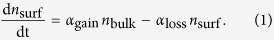


Denoting the initial and steady-state fractions of surface receptors with 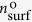 and 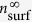, one obtains 
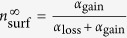
, and the time evolution of the average fraction of surface receptors follows


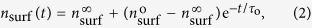


with the characteristic time 
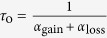
. Thus, 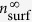 and 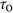 are controlled by the rates *α*_loss_ and *α*_gain_. Indeed, the cycle of receptors in the cell is more complicated; there are more influential parameters involved and the receptor conservation assumption does not hold in general. Nevertheless, as shown in Fig. 2B, Eq. 2 qualitatively reproduces the trends observed in experiments, though the curvature change is not captured.

The previous approach would reflect a situation where the transport of receptors to and from the plasma membrane is neither influenced by exclusion nor by self-amplification. However, if one assumes that binary excluded-volume interactions between receptors have to be considered and/or self-amplification of the receptor transport plays a role, the evolution of the fraction of surface receptors can be roughly described as


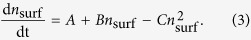


By fitting the free parameters, the above equation captures quantitatively well the *in vivo* observed dynamics over the whole time window (solid line in Fig. 2B).

### Effect of temperature and ligand concentration on KDELR cluster formation

**Figure 3:**
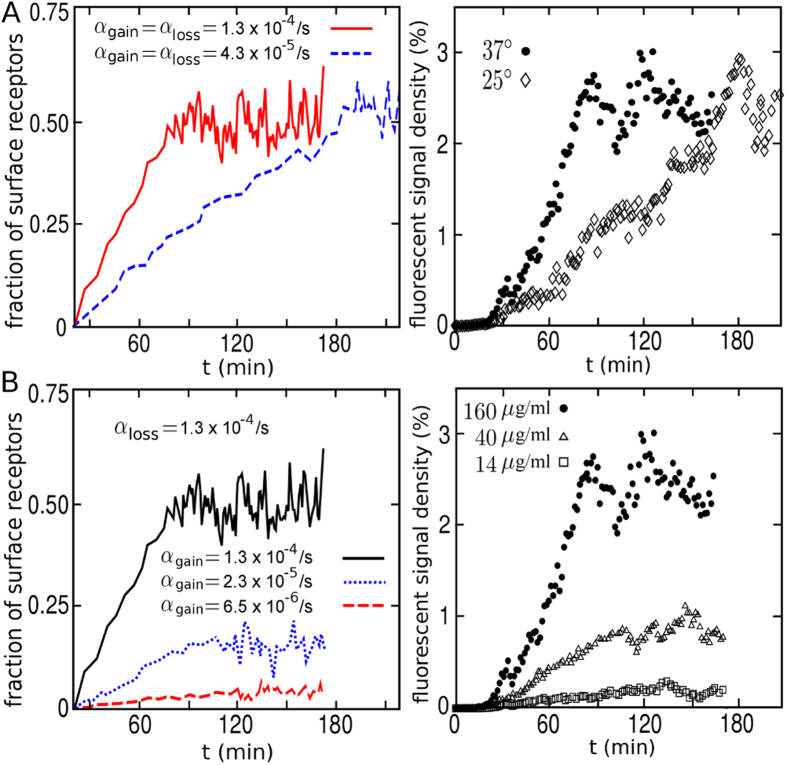
KDELR/cargo clustering is dose- and temperature-dependent. (**A**) Changes in cargo accumulation of eGFP-RTA^HDEL^ at the surface of HeLa cells cultivated at 25 °C or 37 °C. The 3-fold reduced activity in simulations (left) represents the known effect of temperature on intracellular transport processes (e.g. endocytosis and exocytosis). (right) The experimental results at 25 °C and 37 °C. (**B**) Effect of changing the concentration of the model cargo eGFP-RTA^HDEL^ on KDELR/cargo clustering at the plasma membrane. The indicated rates in simulations (left) were adopted to obtain the best fits to the experimental data (right).

Prior to experimentally investigating if temperature changes affect KDELR/cargo clustering at the cell surface and follow the van-‘t-Hoff’sche rules, we assumed that KDELR clustering at 25 °C should slow down by a factor of 2–4 without changing the overall kinetics and shape of the curve. Based on this assumption, we reduced the endocytosis and vesicle arrival rates in simulations by a factor of 3. The results predicted a timely retardation of KDELR membrane cluster formation at 25 °C while the overall saturation level was similar to the one reached at 37 °C (Fig. 3A, left panel). Since the experimental data nicely confirmed the numerical predictions (Fig. 3A, right panel), it can be concluded that KDELR/cargo cluster formation is a temperature-dependent process. It can be also deduced from the minimal analytical model presented in the previous section, that the final saturation level 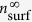 remains invariant under a symmetric scaling of the rates (i.e. *α*_loss_ → *κα*_loss_ and *α*_gain_ → *κα*_gain_), while the characteristic relaxation time is rescaled as *τ*_o_ → *τ*_o_/*κ*.

To determine any effect of cargo concentration on cluster formation at the PM, HeLa cells were treated with different doses of eGFP-RTA^HDEL^; the corresponding results revealed a strict dose-dependency of KDELR/cargo cluster formation at the cell surface (Fig. 3B). In contrast to the impact of temperature, the variation of the saturation level with changing the cargo concentration indicated that the endocytosis and vesicle arrival rates are differently affected, i.e. they scale as *α*_loss_ → *κα*_loss_ and *α*_gain_ → *κ*′*α*_gain_ with *κ* ≠ *κ*′. This is indeed necessary to reproduce the experimental data in simulations (Fig. 3B, left panel). Denoting the steady-state fraction of surface receptors at low and high concentrations with 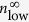 and 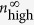 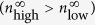, from the minimal theoretical model one finds that 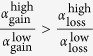, thus, the vesicle arrival rate is more sensitive to the concentration changes than the endocytosis rate. Interestingly, reduction of the saturation level at lower cargo concentrations suggests that mammalian cells can somehow modulate the response depending on extracellular ligand concentration. They are capable to sense the actual concentration of cargo binding and subsequently regulate the total amount of KDELRs at the cell surface. Hence, the combination of experimental data and numerical results provides a first mechanistic insight into KDELR/cargo clustering at the mammalian cell surface.

### Anterograde KDELR transport to preferential plasma membrane arrival sites

The cellular plasma membrane resembles a thoroughly regulated and highly dynamic compartment that contains cell surface micro-domains like lipid rafts or caveolea^30^. It is well documented that plasma membrane receptors such as AchR, EGFR or TGF-*β* are associated with lipid rafts^8^,^31^,^32^ and that preferential receptor cluster formation in distinct micro-domains of the PM provides an important means to regulate downstream signaling as shown for EGFR^8^. Moreover, it has been proposed that T cell receptor pre-clustering at the cell surface contributes to a significant increase in ligand sensitivity and accelerates signaling pathway activation^33^.

**Figure 4:**
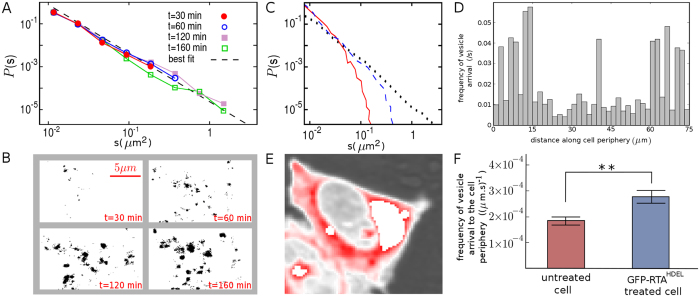
Preferential arrival sites of KDELRs at the plasma membrane. (**A**) The log-log plots of cluster-size distribution *P*(*s*) of eGFP-RTA^HDEL^ (160 *μ*g/ml) treated HeLa cells at the indicated time points. The dashed line corresponds to the best power-law fit *P*(*s*) ~ *s*^−*β*^ with 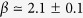. (**B**) Evolution of the receptor clusters at the plasma membrane. A randomly chosen region of the cell surface is shown at different time points (see *Suppl*. *Info*. for the detailed description of the methodology of distinguishing the clusters and obtaining the cluster-size distribution). (**C**) A comparison of the resulting *P*(*s*) from different receptor dynamic scenarios in simulations. The solid, dashed, and dotted lines denote the shape of *P*(*s*) at *t* = 120 min for randomly distributed immobile receptors, aggregation process including lateral diffusion of receptors and nearest-neighbor attraction between them, and preferential attachment process, respectively. (**D**) The frequency of vesicle arrival at a sample cell periphery over a time window of 500 s. (**E**) *In vivo* dynamics of mCherry-ERD2.1. The transfected HeLa cells with mCherry-ERD2.1 were analyzed by CLSM (720 frames/h). The illustrated heat map represents the accumulated fluorescent signals of successive frames. The regions with high traffic load, e.g. around Golgi, are eliminated to provide a more clear color distinction near the cell surface. (**F**) The frequency of vesicle transport near the plasma membrane of untreated or eGFP-RTA^HDEL^ treated cells. The data is averaged over bins of size 10 *μ*m (**P ≤ 0.01, t test).

To understand how KDELR/cargo clustering evolves and whether or not KDELRs are likewise arranged in receptor pre-clusters or micro-domains, cluster size distribution *P*(*s*) of the model cargo eGFP-RTA^HDEL^ was determined at different time points during the clustering process. As shown in Fig. 4A,B, relatively small clusters were visible at early time points, while larger clusters appeared at longer times. A detailed analysis indicated that the growth of the largest cluster eventually stopped after reaching the stationary state. Notably, the cluster size distribution nearly followed a power-law decay *P*(*s*) ~ *s*^−*β*^ with a rather time-invariant exponent *β* ≈ 2 throughout the clustering process, except for the initial transient regime (*t* < 20 min).

The functional form of the cluster size distribution *P*(*s*) indeed implies which of the underlying mechanisms of receptor dynamics is dominant. In Monte Carlo simulations, we first assumed that the target zones for endocytosis and vesicle arrival events are randomly chosen, without allowing the distributed receptors to move on the surface. Next, we examined the main possible scenarios for receptor surface dynamics including lateral diffusion on the membrane^34^,^35^ and receptor-receptor attraction^27^,^28^ (both with the short range of nearest-neighbor sites). Figure 4C shows that none of the resulting aggregation patterns was capable of producing a power-law size distribution; the tails of the resulting distributions rather follow an exponential-like decay. The algebraic form can be recovered under the assumptions that KDELRs have distinct and preferred arrival sites at the plasma membrane and the transport is self-amplified. This was achieved in the following way. The spatial distribution of targeting probability was changed from a uniform to a multiple-peaked Gaussian one. The peaks represent the places where MTs approach the cell cortex and distribute their vesicles, which are supposed to diffuse on the actin filament network until they finally reach the membrane. Additionally, the surface was divided into subdomains obtained by Voronoi tessellation of area around each peak, and a newly generated vesicle choses a target subdomain with a probability proportional to the transport-activity history of the corresponding MT.

Let us consider a rather simple process of receptor aggregation, in which the time evolution of the probability *P*(*s*) of having receptor clusters of size *s* is expressed via the master equation 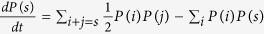. The gain and loss terms on the right-hand side account for creation of *s*-size clusters from the coalescence of two smaller ones of sizes *i* and *j*, and merging of *s*-size clusters with other ones, respectively. Starting from an initial configuration with randomly distributed single receptors, the master equation can be recursively solved to get 
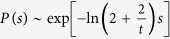
, i.e. *P*(*s*) decays exponentially with a time-dependent exponent. At long times, the slope decreases and *P*(*s*) evolves towards a flat distribution. Our attempts to consider more complicated aggregation scenarios, such as introducing diffusion or aggregation with input, failed to simultaneously reproduce the power-law and time-invariant features of *P*(*s*). In contrast, it can be verified that preferential attachment of receptors to the existing clusters reproduces the experimentally observed distribution. We consider a simple clustering process in which a new receptor is added to the surface at each time step, and it attaches to the cluster of size *s*_*i*_ with a probability 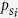 which is proportional to the cluster size, i.e. 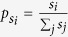. The sum runs over all clusters, thus, reflects the total number of receptors and grows linearly with time. The rate at which *s*_*i*_ changes can be assumed to be proportional to 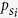



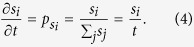


Denoting the initiation time of cluster *i* with *t*_*i*_, one obtains *s*_*i*_(*t*) = *t*/*t*_*i*_. The probability that a cluster is smaller than *s* is given as


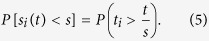


The probability *P*(*t*_*i*_) of initiation at time *t*_*i*_ has a constant probability density with respect to time, i.e. 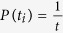. Substituting this into Eq. 5 we get


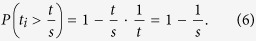


Finally, the cluster-size distribution can be obtained using


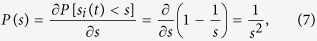


which shows a power-law decay with a time-independent exponent 2, in a remarkable agreement with the experimental results shown in Fig. 4A. Since the receptor trafficking mainly occurs along MTs, vesicle exchange near the PM happens mostly in the vicinity of the regions where MTs approach actin filaments near the cell cortex. Our data are consistent with a feedback mechanism that amplifies receptor transport towards the plasma membrane in the presence of receptor clusters.

Although the *in vivo* observed receptor dynamics is indeed more complicated and also depends on other factors (e.g. membrane thickness, lipid composition etc.) and involves both surface dynamic processes and the membrane-cytoplasm receptor cycle, our numerical and analytical findings suggest that the intracellular transport of vesicles along the microtubule network, which induces preferential zones for vesicle exchange at the PM, crucially controls the clustering at the mammalian cell surface. To experimentally prove this hypothesis, HeLa cells were transfected with mCherry-labeled KDELR1 (Erd2.1) and receptor dynamics was analyzed by live cell imaging (Supplementary Movie S3). By monitoring the frequency of vesicle arrival at the plasma membrane over a time window of 500 s, it is shown in Fig. 4D that anterograde KDELR transport is non-uniformly distributed along the plasma membrane, i.e. there are hot spots on the cell periphery which are targeted more frequently by the arrival of vesicles. The heat map of intracellular KDELR transport in Fig. 4E illustrates the spatio-temporal distribution of KDEL receptors. Notably, a comparison between untreated and eGFP-RTA^HDEL^ treated cells in Fig. 4F indicates an increase in the transport rate in treated cells. Thus, the experimental findings support the numerical predictions and underline the importance of preferential absorption in regulating KDELR cluster formation.

### Microtubule and actin assisted receptor transport and membrane clustering

Active protein transport along the cytoskeleton is mediated by actin filaments and microtubules (MTs). While filamentous F-actin is mainly localized in the cell cortex and involved in cell migration, endocytosis and vesicle-mediated cargo transport, MTs are responsible for dynein/kinesin driven active transport of vesicles and organelles^36^,^37^,^38^.

**Figure 5:**
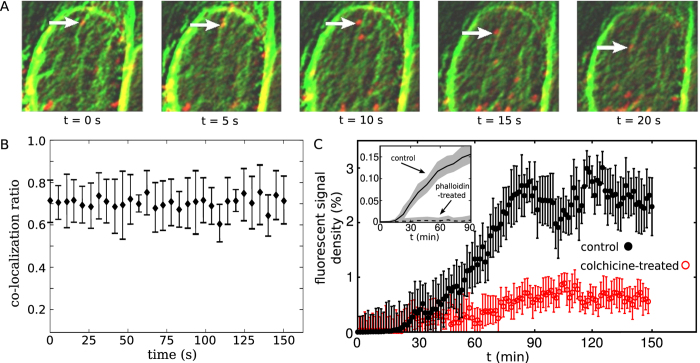
Microtubule-assisted KDELR transport is required for cargo clustering at the cell surface. (**A**) Tracking of single KDELR clusters (red) moving along the microtubule network (green). A sequence of five successive live cell imaging pictures (720 frames/h) of HeLa cells expressing mCherry-tagged Erd2.1 and GFP-tagged *β*-tubulin is shown. The arrows indicate an example of tubulin/KDELR signal co-localization. (**B**) Co-localization of GFP-tubulin and mCherry-Erd2.1. The ratio of correlated tubulin and KDELR pixels of the live cell imaging experiment is shown during 150 s. (**C**) KDELR/cargo cluster formation in colchicine (red) and phalloidin (inset) pre-treated cells (2.5 *μ*M colchicine, 60 min or 10 *μ*M phalloidin, 90 min) after incubation with 160 *μ*g/ml eGFP-RTA^HDEL^. Temporal evolution of the accumulated KDELR/cargo is compared with the untreated control cells. The inset shows a comparison between untreated (solid line) and phalloidin-treated HeLa cells (dashed line).

To verify that the MT-network is involved in intracellular KDELR trafficking, cells were co-transfected with GFP-tagged *β*-tubulin and mCherry-ERD2.1 and KDELR transport along MTs was visualized by CLSM. Despite the limited resolution of live cell imaging, directed transport of KDELR signals along MTs could be clearly observed (see e.g. Fig. 5A), indicating that anterograde receptor transport is indeed MT based. A more quantitative analysis also revealed a relatively high probability of tubulin/KDELR signal co-localization (Fig. 5B). Moreover, we observed that colchicine-mediated inhibition of MT assembly highly affects KDELR dynamics *in vivo* (Supplementary Movie S4A,B) and considerably reduces receptor clustering at the cell surface, see Fig. 5C. We conclude that active receptor transport along MTs is a prerequisite for KDELR/cargo cluster formation at the plasma membrane. It has been shown^34^,^39^,^40^,^41^ that disruption of MTs causes the majority of EGFR or cAMPR clusters to be immobile, or affects the endocytosis of EGFRs; thus, MTs play a crucial role in organizing receptor clusters at the plasma membrane. Interestingly, KDELR mobility at the cell periphery was not completely blocked in colchicine treated cells indicating that cortical actin filaments which are unaffected by the drug are involved in and responsible for the observed KDELR endocytosis from the PM. Consistently, phalloidin-mediated actin inhibition caused a severe impairment of KDELR cluster development at the cell surface (Fig. 5C, inset).

## Discussion

Until recently, cargo recognition by KDEL-receptors has been assumed to mainly occur within the Golgi complex during retrograde transport of soluble ER residents back to the ER^21^,^42^. More recent studies, however, indicated that KDELRs are also responsible for cell surface binding of extracellular ligands such as the neurotrophic factor MANF or the microbial A/B toxin K28^14^,^15^,^16^,^17^. Based on these findings we postulated that a model cargo containing a C-terminal H/KDEL amino acid motif and receptor binding site should likewise bind to cells and, thus, be suitable to track and analyze cargo binding and subsequent cellular responses. Using this approach we now demonstrate that treatment of cells with a fluorescent variant of RTA extended by a C-terminal H/KDEL motif is required and sufficient to promote specific cargo binding and clustering at the mammalian cell surface. Based on the experimental data presented here it can be deduced that the initial binding of eGFP-RTA^H/KDEL^ to the cell periphery occurs within seconds and is immediately followed by the formation of plasma membrane-associated clusters within 20 minutes. Thereafter, cluster development follows an exponential growth and eventually saturates at time points >80 min. During this process, KDELR/cargo clusters are also internalized, however the precise temporal resolution of such endocytosis events has not yet been analyzed and will be subject of future studies. Since all cell binding studies were performed under conditions of natural KDELR *in vivo* expression, and H/KDEL motifs on cellular proteins have solely been attributed to be exclusively recognized by KDELRs, our present data strongly point towards a function of KDELRs at the cell surface. Furthermore, our observation that eGFP-RTA^H/KDEL^ shows a strong increase in toxicity and *in vivo* uptake (data not shown), likewise supports a role of KDELR-mediated cargo/toxin transport from the plasma membrane to the cytosol.

In an approach to characterize the observed clustering at the cell surface after cargo addition, we combined experiments and numerical methods and thereby demonstrate that cargo/KDELR cluster formation over time is a process that equally depends on temperature and cargo concentration. In particular, the later observation strongly points towards a regulated cellular mechanism to respond to an extracellular receptor ligand. Extensive simulations of cluster formation and size distribution indeed indicate that cells are capable to sense external cargo concentration and appropriately adapt the number of receptors at the plasma membrane. External KDEL-cargo addition likewise resulted in a response leading to an increase in anterograde receptor traffic to the plasma membrane. In addition, the lower cargo cluster numbers in the absence of active endocytosis and exocytosis (phalloidin-treatment or 4 °C) indicate that under these conditions significantly less receptor molecules reach the plasma membrane, indirectly supporting our assumption that regulated KDELR transport is important for receptor cluster formation at the cell surface. One of the most striking features of the observed clustering is the power-law decay of cluster-size distribution P(s) with an approximately time-independent slope at longer times. To elucidate the origin of this behavior, we isolated and examined the role of surface dynamic processes such as receptor diffusion and receptor-receptor attraction in simulations, which mainly led to a fast (exponential-like) decay of the tail of P(s). We showed that preferential adsorption of receptors is a natural way to obtain adsorption kinetics and cluster-size distribution. Experimental data indicated that intracellular KDELR transport to the PM occurs along microtubules, and hot spots form in the vicinity of the regions where MTs approach the cell cortex and distribute anterograde arrival of KDELR-containing vesicles at the PM as well as collecting newly formed KDELR/cargo complexes from the cell surface by actin-mediated endocytosis. Both responses represent the dominant mechanisms that control receptor distribution at the cell surface, even if affected by additional factors such as e.g. receptor surface dynamics. We showed that both microtubule network and cortical actin are important key players in the clustering process, and inhibition of MTs or filamentous actin strongly impaired the dynamic clustering at the cell surface. In future studies we intend to use single-molecule cargo tracking in conjunction with high-resolution imaging to further dissect the underlying molecular processes and to identify the cellular components involved in KDELR/cargo cluster formation at the plasma membrane.

## Methods

A detailed description of the experimental procedures and methods can be found in Supplementary Information, including cultivation and transfection of mammalian cells, genetic techniques, affinity purification and immunochemical analysis of PM-localized KDELR1.

## Additional Information

**How to cite this article**: Becker, B. *et al*. Cargo binding promotes KDEL receptor clustering at the mammalian cell surface. *Sci. Rep.* **6**, 28940; doi: 10.1038/srep28940 (2016).

## Supplementary Material

Supplementary Information

Supplementary Movie S1

Supplementary Movie S2

Supplementary Movie S3

Supplementary Movie S4A

Supplementary Movie S4B
