# Supplementary material for: Cargo binding promotes KDEL receptor clustering at the mammalian cell surface
Source: Sci Rep. 2016 Jun 29;6:28940. doi: 10.1038/srep28940 (PMC4926219; doi:10.1038/srep28940)
Supplement: Supplementary Information [file srep28940-s1.pdf]

Supplementary Information to Cargo binding promotes KDEL receptor clustering at the mammalian cell surface

Björn Becker,[1] M. Reza Shaebani,[2] Domenik Rammo,[1] Tobias Bubel,[1] Ludger Santen,[2] and Manfred J. Schmitt[1]

[1]*Molecular and Cell Biology, Department of Biosciences (FR 8.3) and Center of Human and Molecular Biology (ZHMB), Saarland University, D-66123 Saarbrücken, Germany*

[2]*Department of Theoretical Physics, Saarland University, D-66041 Saarbrücken, Germany*

MATERIALS AND METHODS

Cultivation and transfection of mammalian cells

HeLa (ATCC number CCL-2), HEK-293T (Invitrogen) and RAW-Blue cells (Invitrogen) were cultivated at 37°C in DMEM medium (Gibco) supplemented with 10% fetal bovine serum (Biochrom) and 1% penicillin/streptomycin (PAA) in a humidified environment with 5% CO_2. Transfections were performed with plasmid vectors using FuGENE®HD (Promega) according to the manufacturer's instructions.

Vector construction and genetic techniques

Standard molecular manipulations were performed as described[1]. *E. coli* TOP10 was used for cloning (see Supplementary Table 1). Using pRS316-RTA as template, $RTA_{(His)6}$, $RTA_{(His)6HDEL}$, and $RTA_{(His)6KDEL}$ were amplified by conventional PCR with primers listed in Supplementary Table 2. Each DNA fragment was subcloned into pSTBlue-1 (Novagen), routinely sequenced, and cloned as *BamH*I/*Hind*III fragment into pET24a$^{(+)}$ (Novagen). In a second step, amplified mammalian enhanced GFP was integrated via *Nhe*I/*Bam*HI upstream of the RTA variants to obtain the expression vectors pET-eGFP-RTA, pET-eGFP-RTA^{HDEL}, and pET-eGFP-RTA^{KDEL}, respectively. All constructs were subsequently transformed into *E. coli* BL21 (DE3) (Stratagene) and after the cells had reached an $OD_{600}{\sim}0.8-1$, expression of each RTA variant was induced in the presence of 1 mM IPTG for 5 h at 20°C. Cells were harvested, washed twice with sterile water and resuspended in binding buffer (500 mM NaCl, 10 mM imidazol and 20 mM KH_2PO_4) for subsequent sonification. Cell debris was removed and the supernatant was collected for Ni^{2+}-NTA affinity chromatography. Sonicated supernatants were immediately used for SDS-PAGE and Western analysis. Erd2.1mCherry was constructed by SOE-PCR with primers as listed in Supplementary Table 2, subcloned in pcDNA3.1 (Invitrogen) via *Xba*I/*Apa*I and finally integrated into pCDNA5-FRT/TO (Invitrogen) via *BamH*I/*Apa*I. Erd2.1mCherry (mCherry fused to the C-terminus of Erd2.1) as well as Erd2.1-BBS-cmyc were constructed by SOE-PCR with primers as listed in Supplementary Table 2. Erd2.1mCherry was subcloned in pcDNA3.1 (Invitrogen) via *Xba*I/*Apa*I and finally integrated into pCDNA5-FRT/TO (Invitrogen) via *BamH*I/*Apa*I to obtain pErd2.1mCherry. Erd2.1-BBS-cmyc was integrated via *Xho*I/*Not*I into pmCherry-N1 (Clontech) to obtain pErd2.1-BBS-cmyc.

Affinity purification and immunochemical analysis of KDELR model cargo

Sonicated supernatants of *E. coli* clones expressing $(His)_6$-tagged eGFP-RTA variants were purified via Ni^{2+}-NTA affinity chromatography as previously described[2]. Eluted protein fractions were desalted and equilibrated in PBS (pH 7.4). After concentration through 10 kDa cut-off spin columns (Sartorius, Viva Spin 20), purified proteins were stored at 4°C. Western analysis was employed to verify protein expression. Total protein SDS-PAGE was performed under non-reducing conditions in 15% Tris-Tricine gels[3]. Semi-dry blotting onto PVDF membranes was carried out in transfer buffer (25 mM Tris, 190 mM glycin, 0.1% SDS, 20% methanol). Blots were either incubated with antibodies against the cytotoxic A-subunit of RTA and HRP-coupled anti-sheep or analyzed under UV light (362 nm) to detect GFP fluorescence. After incubation with Western lightning Plus ECL (PerkinElmer), signals were detected with ChemiDoc XRS (BioRad). Antibody dilutions are described in Supplementary Table 3.

Cell viability assay and binding studies

To determine biological activity of the purified RTA variants, $1{\times}10^5$ HeLa cells were grown in 24 well plates (DMEM) at 37°C and 5% CO_2 for 24 h. Thereafter, cells were incubated in the presence of the purified RTA variants

(160 μg/ml) for another 48 h. Subsequently, cell viability was determined in an XTT-based viability assay (TOX2, Sigma) according to the manufacturer's instructions. For KDEL-cargo binding studies, 1×10^5 HeLa, HEK-293T or RAW-Blue cells were seeded in 60μ-ibiTreat-dishes (Ibidi) 24 h prior the binding experiment and subsequently treated with 160 μg/ml eGFP-RTA$^{H/KDEL}$ or eGFP-RTA (negative control). After 5 min incubation at 37°C, toxin solution was removed (10 washing steps with DMEM medium w/o phenol red) and cargo binding was analyzed via confocal laser scanning microscopy (CLSM).

Bungarotoxin binding assay

HeLa cells transfected with pERD2.1-BBS (KDELR1) were washed with PBS (pH 7.4) and resuspended in DMEM (w/o phenol red, FCS and penicillin/streptomycin) containing 10 μg/ml AlexaFluor 488 conjugated α-bungarotoxin (Btx) (LifeTechnologies). After 1 h at 37°C, cells were washed three times with PBS and fixed in 3% paraformaldehyde. After five subsequent washing steps, Btx binding was analyzed via CLSM using a Zeiss LSM 510 META.

Immunostaining

For immunofluorescence of HeLa cells transfected with pERD2.1-BBS-cmyc, cells were washed with PBS, fixed with 3% paraformaldehyde and permeabilized with 0.5% Triton-X-100. To stain cmyc-tagged Erd2, cells were blocked in 1% BSA in PBS for 1 h, incubated for 2 h with anti-cmyc, washed six times with PBS (pH 7.4) and incubated with fluorescein-labeled anti-mouse IgG. For giantin double staining, the procedure was repeated with anti-giantin and with PE-labeled anti-rabbit IgG. Image analysis was performed with a Biozero BZ-8000 fluorescence microscope (Keyence) under standard settings.

Immunofluorescence analysis on non-permeabilized cells

HeLa cells (1×10^5) were seeded on cover slips (12 mm), cultivated for 18 h and incubated for 1 h with 160 μg/ml eGFP-RTA^{HDEL} or eGFP-RTA at 37°C. To block endocytosis, cells were subsequently cooled on ice for 20 min. After ten washing steps with PBS (pH 7.4), cells were fixed with 3% paraformaldehyde at 4°C and blocked in 1% BSA in PBS for 1 h at RT. To stain cell-bound GFP-RTA variants, cells were incubated for 2 h with anti-GFP, washed six times with PBS (pH 7.4) and incubated with PE-labeled anti-mouse IgG. After six additional washings, image analysis was performed with a Biozero BZ-8000 fluorescence microscope (Keyence) under standard settings.

Cell surface biotinylation

HeLa cells were plated at 1.5×10^6 cells per 100 mm-dish and allowed to grow for 18 h. After transient transfection with pCDNA5-Erd2.1-V5, cells were grown for additional 48 h and surface biotinylation was carried out with a cell surface protein isolation kit (Pierce) according to the manufacturer's instructions. In brief, cells were cooled on ice, washed three times with ice-cold PBS (pH 7.2), and labeled for 40 min in a Sulfo-NHS-SS-Biotin solution (0.25 mg/ml in PBS) at 4°C. The biotinylation reaction was quenched for 15 min at 4°C, cells were washed three times with cold TBS buffer, resuspended in 300 μl lysis buffer containing protease inhibitor (Roche) and subsequently lysed by sonification followed by 30 min incubation on ice. After centrifugation for 10 min at 15.000 rpm, an aliquot of the cell lysate (50 μl) was removed as input control and the remaining cell lysate (250 μl) was used for streptavidin pull-down performed overnight at 4°C with end-over-end rotation. Samples were washed five times with 1 ml of protease inhibitor containing wash buffer and eluted within 1 h at room temperature in 250 μl 3× SDS buffer containing 50 mM DTT and 5% 2-mercaptoethanol. Input and membrane fractions (20 μl each) were separated by SDS-PAGE and subjected to western analysis. By using antibodies against β-actin, the cell integrity was checked during the labeling step. α1-Na/K-ATPase antibodies served as positive control to confirm success of Sulfo-NHS-SS-Biotin labeling.

Determination of KDELR endocytosis

KDELR endocytosis in HeLa cells was assessed and determined by a modified cell surface biotinylation approach in which an additional cell sample was washed three times with pre-warmed DMEM medium (10% FCS, 1% Pen/Strep)

and incubated for 30 min at 37°C to trigger receptor internalization. Thereafter, cells were washed three times with ice-cold TBS buffer and shifted to 4°C to stop KDELR internalization. Control samples were kept on ice during the entire procedure and served as (i) control for the total amount of cell surface proteins at time 0 and (ii) stripping control. Endocytosis sample and stripping control were treated with ice-cold TBS containing 40 mM Tris (2-carboxyethyl) phosphine (TCEP, 2×15 min, 4°C) to remove residual biotin from the cell surface. Internalized biotinylated proteins are protected from this stripping step and remain biotinylated. After cell lysis, biotinylated proteins were isolated by streptavidin pull-down and ERD2.1-V5 was detected and quantified by immunoblotting. Stripping efficiency was determined as follows, and only experiments with a stripping efficiency of > 95% were used for further analysis:

$$\text{stripping efficiency} = [1 - (\text{stripping control}/\text{total})]\times 100\%. \quad (1)$$

Live cell imaging

HeLa cells (5×10^4 to 1×10^5 cells) were seeded in 60μ-dishes and cultivated for 24 h. Thereafter, cells were washed two times with PBS (pH 7.4), cultivated in DMEM (w/o phenol red, 10% FCS) and analyzed by CLSM. In each experiment, time resolution is expressed as frames per hour (frames/h) and indicated in each Figure legend. To study cargo-induced cluster formation at and endocytotic internalization from the cell surface, cells were treated with eGFP-RTA-His^{HDEL} (160 μg/ml) and monitored for at least 3 h at 37°C and 5% CO_2. RTA variants lacking a KDELR binding site served as negative control. For analyzing the effect of cargo concentration on cluster development, different doses (14, 40, and 160 μg/ml) were applied to HeLa cells and cluster development was analyzed for at least 3 h at 37°C and 5% CO_2. In temperature experiments, KDELR/cargo clustering was investigated at 25°C and 37°C under the same conditions. To determine KDELR dynamics, 1×10^5 HeLa cells were seeded in 60μ-dishes and pcDNA5 FRT/TO-Erd2.1-mCherry transfection was performed 24 h later. After an additional incubation for 48 h, cells were washed twice with PBS (pH 7.4) and monitored in DMEM (w/o phenol red, 10% FCS) for at least 3 h.

Colchicine treatment and microtubule staining

HeLa cells (1×10^5) were seeded in 60μ-dishes, transfected with pcDNA5 FRT/TO-Erd2.1-mCherry 24 h later and incubated for 48 h at 37°C and 5% CO_2. After washing with PBS (pH 7.4), cells were incubated in DMEM (w/o penol red, 10% FCS) supplemented with 2.5 μM colchicine for 1 h and KDELR dynamics was monitored via CLSM. In cargo clustering experiments, HeLa cells were pre-treated for 1 h with 2.5 μM colchicine before DMEM medium containing eGFP-RTA^{HDEL} (160 μg/ml) was applied to the cells. Monitoring was performed at 37°C and 5% CO_2 for at least 3 h. For β-tubulin staining, pcDNA5 FRT/TO-Erd2.1-mCherry transfected HeLa cells were treated with CellLight®Tubulin-GFP reagent (Thermo Fisher Scientific, BacMam system) according to the manufacturer's instructions. Staining was performed 24 h before analysis by CLSM.

Phalloidin treatment

HeLa cells (1×10^5) were seeded in 60μ-dishes and incubated for 24 h at 37°C and 5% CO_2. After washing with PBS (pH 7.4), cells were pre-treated for 90 min in DMEM (w/o penol red, 10% FCS) supplemented with 10 μM phalloidin before eGFP-RTA^{HDEL} (160 μg/ml in DMEM medium) was added to the cells. Monitoring was performed at 37°C and 5% CO_2 for at least 2 h.

Confocal microscopy

Live cell imaging of eGFP and/or mCherry fusion proteins was performed by confocal fluorescence microscopy using a Zeiss LSM 510 META (Nikon PlanApo 63x NA 1.4 oil immersion lens, 488 nm excitation, 2 – 2.5% argon laser power, HFT 488 and NFT 490 beam splitter, BP 500-530 filter; 514 nm excitation, 4% - 6% argon laser power, HFT 514 and NFT 545 beam splitter, LP 560 filter) or Leica TCS SP8 X (HC PL APO CS2 63x/1.4 oil, Scan speed 700 HZ, 586 nm excitation, 1.3 – 1.6% white light laser power, HPD filter 599nm-691nm). All images within a single experiment were collected using the same laser power and pinhole size and processed in an identical manner.

Evaluation of cluster-size distribution

Color images were extracted from the original videos and then converted to gray scale ones such that the intensity of each pixel ranged from 0 (black) to 255 (white). Then, the intensity field was smoothened by means of the best anisotropic Gaussian fit which allowed to determine background noise around each local intensity peak. Noise was subtracted using a threshold ratio of 0.06 between the background intensity and the local peak. The resulting islands were identified as the receptor clusters. We carefully checked that our results are robust against the chosen parametrization of the smoothening procedure. Next, the pixel intensity data was converted to a 2D array of binary data (1:containing a receptor, 0:empty), and Hoshen-Kopelman algorithm was used to identify the clusters and their respective size. Finally, the cluster-size distribution $P(s)$ was obtained by logarithmic binning of the cluster-size range.

---

[1] Sambrook J, Maniatis T & Fritsch EF. *Molecular cloning: a laboratory manual.* Cold Spring Harbor Laboratory Press, Cold Spring Harbor, New York (1989) .
[2] Becker B & Schmitt MJ. Adapting yeast as model to study ricin toxin a uptake and trafficking. *Toxins* **3**, 834-847 (2011).
[3] Schägger H & von Jagow G. Tricine-sodium dodecyl sulfate-polyacrylamide gel electrophoresis for the separation of proteins in the range from 1 to 100 kDa. *Anal Biochem* **166**, 368-379 (1987).

Supplementary Movie S1: Dynamics of cargo binding to HeLa cells treated with eGFP-RTA^{HDEL} (160 μg/ml). Movie shows a part of the complete experiment illustrated in Fig. 1F and starting 30 min after toxin treatment (45 frames/h).

Supplementary Movie S2: Dynamics of cargo binding to HeLa cells treated with eGFP-RTA^{HDEL} (80 μg/ml). CLSM analysis started 6 h after cargo application (60 frames/h).

Supplementary Movie S3: KDELR dynamics in HeLa cells transfected with pCDNA5-Erd2.1-mCherry. Cells were transfected 48 h before CLSM analysis (720 frames/h).

Supplementary Movie S4: KDELR dynamics in colchicine-treated HeLa cells transfected with pCDNA-Erd2.1-mCherry. After 48 h, cells were treated with (A) or without (B) 2.5 μM colchicine for 1 h and subsequently analyzed via CLSM (720 frames/h).

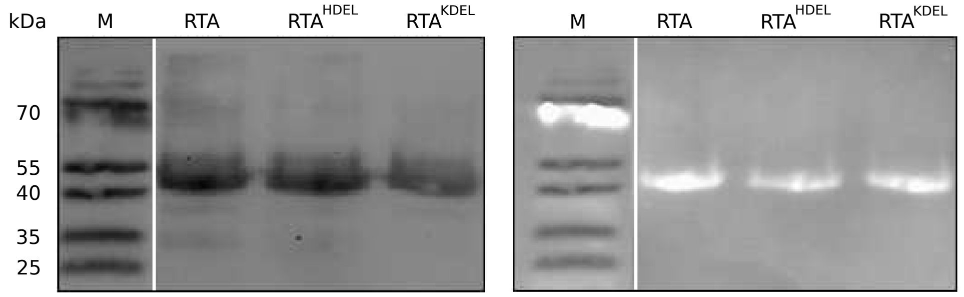

Supplementary Figure S1: Western analysis of recombinant eGFP-RTA variants after Ni^{2+}-NTA affinity purification. Western blots were probed with anti-RTA and anti-sheep-HRP (left) or visualized under UV-light (right) to detect the indicated eGFP-RTA fusion protein (M denotes protein ladder that was run on the same gel but cut between the sample lanes).

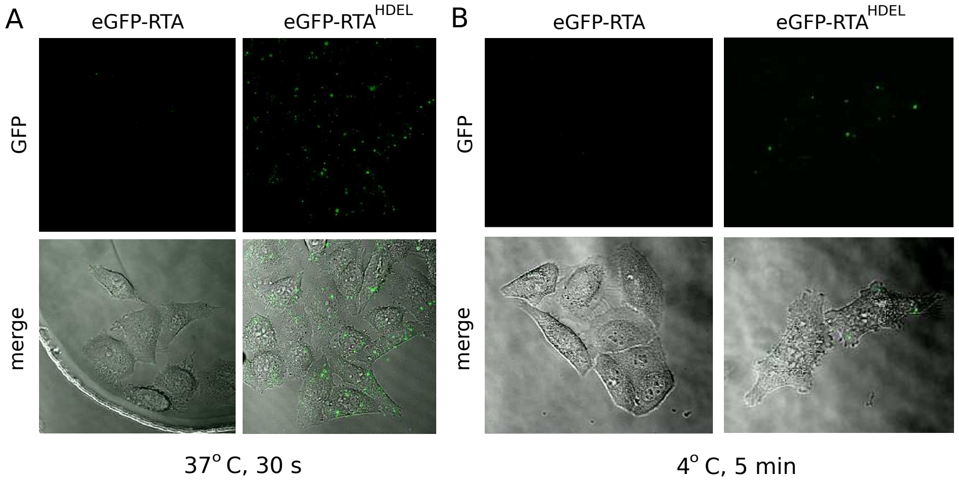

Supplementary Figure S2: (A) Fluorescence microscopy of HDEL-cargo binding at the cell surface. HeLa cells were treated with 160 μg/ml eGFP-RTA^{HDEL} or eGFP-RTA for 30 s at 37°C and cargo binding was analyzed after ten washing steps. (B) Same as (A), except that HeLa cells were initially incubated for 20 min at 4°C to block endocytosis and thereafter treated with 160 μg/ml of the indicated eGFP-RTA variants for 5 min.

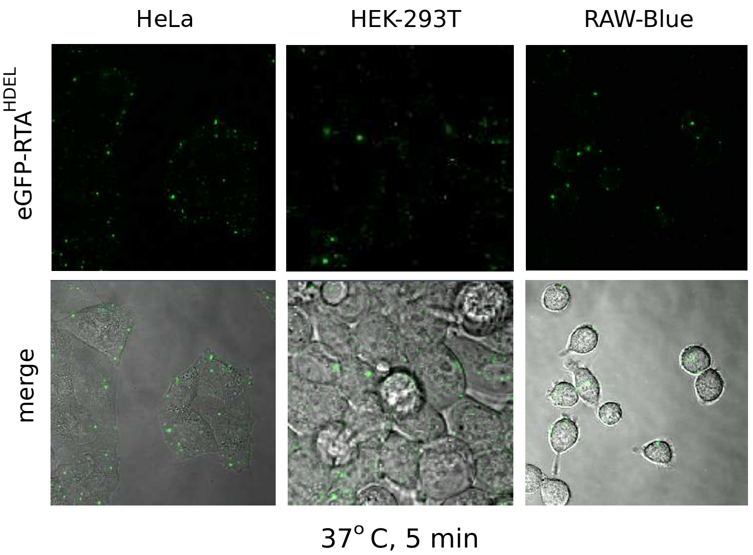

Supplementary Figure S3: Fluorescence microscopy of HDEL-cargo binding at the cell surface of the indicated cell lines. In each case, cells were treated with 160 μg/ml eGFP-RTA^{HDEL} for 5 min at 37°C and analyzed for cargo binding via CLSM after ten washing steps.

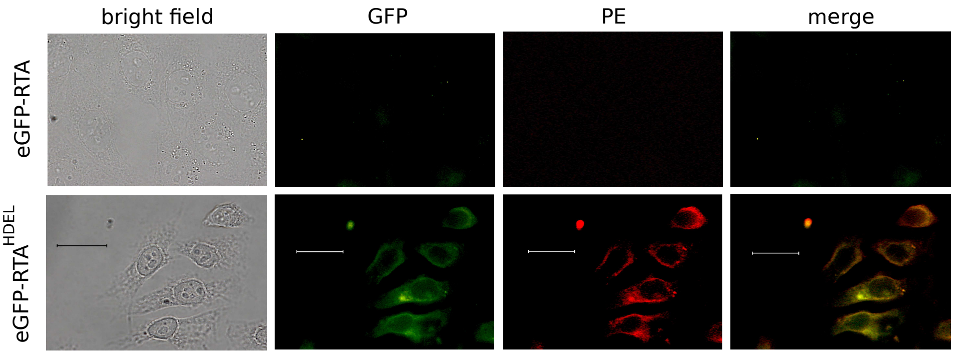

Supplementary Figure S4: Cargo binding and cluster formation at the plasma membrane. Immunostaining of eGFP-labeled HDEL-cargo binding was performed on non-permeabilized cells treated with 160 μg/ml eGFP-RTA^{HDEL} or eGFP-RTA for 1h at 37°C and subsequently fixed with 3% paraformaldehyde. Extracellular cargo binding was visualized by indirect immunofluorescence microscopy using anti-GFP and anti-PE as primary and secondary antibody, respectively. Co-localization of cell-bound cargo (red, PE) and GFP-fluorescence (green, GFP) at the plasma membrane is shown in yellow (merge) (Scale bar 10 μm).

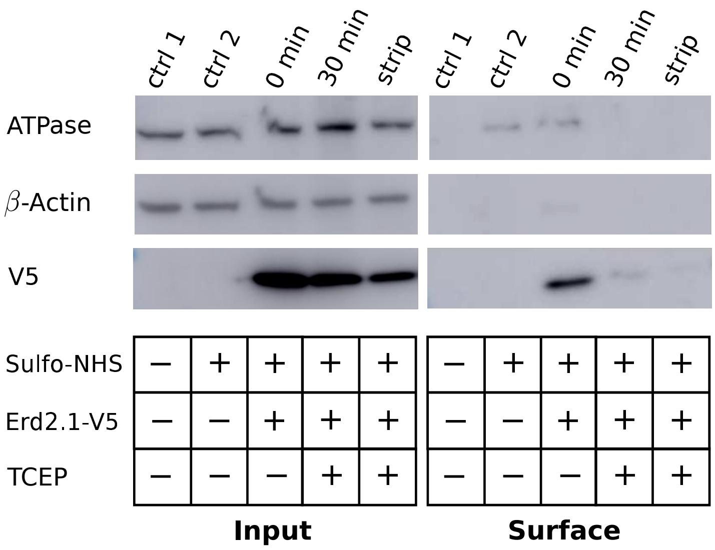

Supplementary Figure S5: Determination of KDELR endocytosis from the cell surface. HeLa cells were transiently transfected with KDELR1 (Erd2.1-V5 (+)) or an empty vector (-) and cultivated for 48 h. Cell surface proteins were biotinylated by treatment with (+) or without (-) Sulfo-NHS-SS-Biotin. KDELR1 endocytosis was triggered by incubating the cells for 30 min at 37°C and biotinylated proteins were subsequently stripped from the cell surface by treatment with TCEP (+). An additional control (0 min) not treated with TCEP (-) was continuously kept on ice and used to determine the total amount of receptors at the cell surface. Stripping control (strip) served to calculate the stripping efficiency (98.5% in this experiment), the membrane fraction (surface, 30 min) represents the amount of KDELRs internalized from the cell surface; KDELR1 expression was determined by immunoblot probed with anti-V5.

Supplementary Table 1. *E. coli* strains used in this study

E. coli strain	Genotype	Reference
TOP10	F′ mcrA Δ(mrr-hsdRMS-mcrBC) Φ80lacZΔM15 ΔlacX74 recA1 deoR araD139 Δ(ara-leu)7697 galU galK rpsL (Str^R) endA1 nupG	Invitrogen
BL21 (DE3)	F′ ompT gal dcm lon hsdSB($r_B^- m_B^-$) λ(DE3 [lacI lacUV5-T7 gene 1 ind1 sam7 nin5])	Biomol

Supplementary Table 2. Primers used in this study (restriction sites are shown in lower case letters)

Primer	5′-3′ Sequence
5′ Erd2.1	gaattctctagaATGAATCTCTTCCGATTCCTGGGAGAC
5′ Erd2.1mCherry-SOE	GGAAGAAGTTGAGTTTGCCGGCAATGGTGAGCAAGGGCGAGGAG
3′ mCherry	ggatccgggcccTTACTTGTACAGCTCGTCCATGCCG
3′ Erd2.1mCherry-SOE	CTCCTCGCCCTTGCTCACCATTGCCGGCAAACTCAACTTCTTCC
5′ RTA	gaattcggatccATGATATTCCCCAAACAATACCCAATTATAAACTTTACC
3′ $RTA_{(His)6}$	aagcttgtcgacTTAATGATGATGATGATGATGAAACTGTGACGATGGTGGAGGTGC
3′ $RTA_{(His)6}^{HDEL}$	aagcttgtcgacTTACAGTTCATCATGATGATGATGATGATGATGAAACTGTGACGATGGTGGAGGTGC
3′ $RTA_{(His)6}^{KDEL}$	aagcttgtcgacTTACAGTTCATCTTTATGATGATGATGATGATGAAACTGTGACGATGGTGGAGGTGC
5′ eGFP	tctagagctagcATGGTGAGCAAGGGCGAGGAGC
3′ eGFP	gtcgacTTAggatccCTTGTACAGCTCGTCCATGCCG
5′ Erd2.1-BBS	gctagcctcgagATGAATCTCTTCCGATTCCTGGGAGAC
5′ SOE-Erd2.1-BBS	ATGAGATACTACGAATCTTCTCTGAAATCTTACCCAGATCCTCTGGAGATCCTCTGGACCTTCTCC
3′ SOE-Erd2.1-BBS	ATCTGGGTAAGATTTCAGAGAAGATTCGTAGTATCTCATGGTGAAGTCATGATTGACCAGGAACG
3′ Erd2.1-BBS-cmyc	gcggccgctcgacgaattcCTAcagatcttcttcagaaatcagcttctgttcTGCCGGCAAACTCAACTTCTTCC

Supplementary Table 3. Antibodies used in this study

Antibody	Dilution	Source
anti-RTA, sheep	1 : 1,000	Lynne Roberts
anti-sheep-HRP	1 : 13,000	Sigma (A3415)
anti-β-Actin, mouse	1 : 1,000	Abcam
anti-α1 Na/K ATPase, mouse	1 : 1,000	Abcam
anti-V5, mouse	1 : 1,000	AbD SeroTec
anti-PE, mouse	1 : 50	Sigma
anti-GFP, mouse	1 : 50	Roche
anti-cmyc, mouse	1 : 400	Roche
anti-FITC, mouse	1 : 100	Merck
anti-Giantin, rabbit	1 : 200	Abcam
anti-PE, rabbit	1 : 100	Sigma
